# Learning by chance: Investigating gaps in transgender care education amongst family medicine, endocrinology, psychiatry and urology residents

**DOI:** 10.36834/cmej.53009

**Published:** 2020-08-06

**Authors:** Raymond Fung, Claire Gallibois, Alexandre Coutin, Sarah Wright

**Affiliations:** 1 Michael Garron Hospital, University of Toronto, Ontario, Canada; 2 Royal College of Surgeons in Ireland, Dublin, Ireland; 3 University of Ottawa, Ontario, Canada; 4 University of Toronto, Ontario, Canada

## Abstract

**Background:**

The transgender (trans) population is one of the most underserved in health care. Not only do they face discrimination and stigma from society as a whole, they also have difficulty accessing transition-related care, leading to adverse outcomes such as suicide. We aimed to increase understanding on how our current postgraduate education system contributes to a lack of care for trans patients.

**Methods:**

Our study consisted of 11 semi-structured interviews conducted in 2016 with residents in the following specialties: family medicine (3), endocrinology (3), psychiatry (3), and urology (2). We used Framework Analysis to qualitatively analyze our data.

**Results:**

Residents described a lack of trans care education in the core curriculum, in part due to a lack of exposure to experts in this area. They also expressed discomfort when dealing with trans patients, due to inexperience and lack of knowledge. Furthermore, residents in each specialty had false assumptions that other specialties had sufficient knowledge and expertise in trans care.

**Discussion:**

This study highlights how the lack of teaching and clinical experiences with trans patients during residency contributes to the poor access to healthcare. By systematically embedding trans care in the curriculum, medical education can play a prominent role in addressing the healthcare disparities of this underserved population.

## Abstract (fr)

**Contexte:**

La population transgenre (trans) est l’une des plus mal desservies en matière de soins de santé. Non seulement les transgenres font-ils face à de la discrimination et stigmatisation provenant de la société en général, mais ils ont aussi de la difficulté à accéder aux soins liés à leur transition, ce qui mène souvent à des résultats particulièrement néfastes tel le suicide. Notre but était de mieux comprendre comment le système d’enseignement postgradué actuel contribue au manque de soins pour les patients trans.

**Méthodes:**

Notre recherche consistait en onze entrevues semi-structurées effectuées en 2016 auprès de résidents des spécialités suivantes : médecine familiale (3), endocrinologie (3), psychiatrie (3) et urologie (2). Nous avons utilisé une méthode »Framework Analysis » pour analyser qualitativement nos données.

**Résultats:**

Les résidents ont décrit un manque de formation en soins pour les transgenres dans le cursus de base; celui-ci étant causé en partie par un manque d’exposition aux experts dans ce domaine. Ils ont aussi exprimé un inconfort au moment de prendre en charge les patients trans qu’ils ont expliqué par leur inexpérience et manque de connaissances. De plus, les résidents de chaque spécialité entretenaient la fausse idée que les autres spécialités possédaient la connaissance et l’expertise nécessaires en soins trans.

**Discussion:**

Cette étude met en lumière comment le manque d’enseignement et d’expérience clinique auprès des patients trans durant la résidence contribue à l’accès très limité de ces derniers aux soins de santé. En intégrant systématiquement les soins trans dans le cursus, la formation médicale peut jouer un rôle majeur lorsqu’il s’agit de combler les disparités en soins de santé de cette population mal desservie.

## Introduction

Though only 0.5-1.2% of the population identify as transgender (trans),^1^ they are underserved in the healthcare system. Inadequate access to transition-related healthcare can result in severe consequences, such as unsafe self-administered hormone therapy, self-performed surgery, or even suicide.^2-7^ Traditionally, psychiatrists have been the gatekeepers to medical and surgical therapy for trans people, which in itself constitutes a substantial barrier to accessing care.^8^ Trans people also have healthcare needs unrelated to their trans status, *ergo* all medical specialties should be prepared to treat this population. However, research has shown that healthcare encounters do not always meet the needs of patients within this population, with some experiencing discomfort arising from a perceived lack of knowledge on the physician’s part,^9^ discrimination, and stigma,^10^ or receiving inadequate or inappropriate care.^11^

Physicians’ lack of preparation and comfort level, stemming from inadequate training, contribute to the ongoing and problematic health disparities experienced by trans people.^12^ Recent studies have indicated that physician trainees feel unprepared to handle the healthcare needs of trans patients, despite a willingness and desire to learn.^13^ Not surprisingly, provider education levels related to trans care have been associated with higher levels of patient comfort and increased access to services,^11^^,^^14^^,^^15^ indicating that medical education can effect positive change. Thus, medical education is well positioned to play a role in addressing the difficulties faced by trans people when they engage with the healthcare system. Failure to do so will likely contribute to ongoing health disparities.

There have been attempts to study LGBT (lesbian, gay, bisexual and transgender) content in medical school,^16-18^ but many of these studies are not focused on trans care and do not extend to postgraduate curriculum as a whole. Other studies have looked at individual residency programs,^19-21^ however, they have not examined residency experience in transgender care holistically across multiple training programs. During residency, trainees learn the specifics of a specialty, but also experience a socialization process in which they learn the *culture* of their specialty.^12^^,^^22^ Scholars have critiqued a lack of curriculum time devoted to trans care in medical school contributing to a culture of ‘silence and discrimination.’^12^ In addition to what is explicitly included in curriculum, informal socialization of attitudes and values (the hidden curriculum.^23^^,^^24^) may be just as important, We feel that a qualitative approach is best suited to improve our understanding of the lived experience of resident physicians and to uncover aspects of the hidden curriculum.

Our group previously conducted a survey of residents’ views on the adequacy of their trans care education, their interest in participating in trans care, and their expectations of competence in trans care by the end of their residency.^12^ Despite a substantial portion of residents expressing interest in learning about trans care, most of them rated their educational experience as inadequate. They also felt that by the end of their residency they would not be competent to provide trans care. We, therefore, undertook this qualitative study to understand the experiences of residents across a variety of specialties likely to provide care to trans people, to identify systemic issues that contribute to the difficulties trans people face when accessing care, and to uncover possibilities for educational interventions to address these gaps. We explored resident training experiences related to caring for trans people, their feelings of preparedness, and how they navigated challenges that arose in practice in four different specialties: endocrinology, urology, psychiatry, and family medicine.

## Methods

### Study design

We obtained ethics approval from the University of Toronto Research Ethics Board (Protocol #32996, May 13, 2016). In 2016, we conducted qualitative interviews with 11 residents in four different specialties to maximize the variation in our sample.^25^ We recruited participants following a related survey study in which all residents from endocrinology, urology, family medicine, and psychiatry from the University of Toronto (556) were invited to participate. We selected these specialties due to their major role in facilitating medical and surgical transition-related care. Out of 325 survey respondents, 85 indicated their willingness to be contacted for future studies related to trans care education, resulting in 11 interviews. Our recruitment efforts focused on residents in more senior years of their residency program to maximize the possibility of exposure to trans patients and teaching in the clinical setting.

### Research team and reflexivity

In qualitative research, it is important to be reflexive; or to understand how the background knowledge and experiences of each team member influences the study.^26^ Dr. Raymond Fung, the principal investigator, is an endocrinologist at Michael Garron Hospital specializing in trans care and has been involved in developing medical education initiatives to address the lack of training in this area. At the time of data collection, Alexandre Coutin (AC) was a second-year medical student with an interest in LGBTQ health issues and medical education. He conducted all of the interviews and did not know any of the participants. AC is a queer cisgender male student and a longstanding ally to the trans community. Claire Gallibois (CG) who contributed to the data analysis, was a fourth-year medical student at the Royal College of Surgeons in Ireland who joined the project via a summer research program held at Michael Garron Hospital. Dr. Sarah Wright (SW) is a PhD Scientist at Michael Garron Hospital with an interest in how structural hierarchies reproduce healthcare and education inequities. SW provided conceptual and methodological guidance to the project.

### Participants

Using a maximum variation sampling strategy,^25^ we interviewed 11 residents: three from endocrinology (total number of residents in the program: 10), three from family medicine (total number of residents in the program: 386), three from psychiatry (total number of residents in the program: 154), and two from urology (total number of residents in the program: 17). The three endocrinology residents were at the end of their PGY 4 year (of a five-year program). The three family medicine residents were in PGY 2, the last year of their program. The two urology residents were in PGY3 and PGY 5, and the psychiatry residents were in PGY 3, 4, and 5 respectively. Participants had all attended undergraduate medical school in Canada. Even though gender identity was collected in an open-ended manner, all participants identified as cisgender, with seven identifying as male, and four as female.

Interviews were semi-structured, following a topic guide that allowed the interviewer to ask additional questions as they arose. Discussions covered areas including experiences and encounters with trans patients, feelings of preparedness to care for this patient population, and how uncertainty was handled when caring for trans patients. Each interview was approximately one hour long. Participants received an honorarium in appreciation for their time.

### Analysis

All interviews were audio recorded and transcribed. Interviews were analyzed using Framework Analysis, a type of thematic analysis, using the following steps: familiarization, creating a thematic framework, indexing, charting, mapping, and interpretation.^27^ All members of the research team reviewed the transcripts and the team agreed upon an initial thematic framework. CG applied the thematic framework to the transcripts (indexing, charting, and mapping), while checking in with the research team on a regular basis to refine the framework and resolve coding discrepancies.

## Results

The results focus on three main themes brought up by participants: learning about trans care by chance, navigating clinical experiences with trans patients, and uncertainty and assumptions made about which specialties should provide care to trans patients.

### Learning by chance

Participants in all specialties cited a lack of both formal teaching and clinical encounters with trans patients, with some participants citing no clinical encounters at all during residency. Participants noted a paucity of faculty with clinical expertise in trans care:

I don't have someone in the faculty that is a guru in the area [...] I mean, I could speak to some of our social workers […] but I don't know about, you know, clinical education, who would be a good, sort of, role model. I can't think of any. P9, Family Medicine

In psychiatry, one resident took it upon himself to become a trans education expert, in the absence of educators who could fulfill this role, serving as a key resource for residents who have questions about trans care:

He has a very strong interest in LGBTQ issues and specifically transgender community. So he’s sort of like our go-to person [point person] if we have questions. P7, Psychiatry

We were able to interview this individual, who spoke of his self-motivation to seek out experiences in trans care since they were not provided in the standard curriculum:

I did an elective with a psychiatrist […] a few days a week and specifically worked with LGBTQ populations and new immigrants. That was mostly how I got my experience. Otherwise nothing really through regular clinical encounters. P11, Psychiatry

While the participant recognized the appreciation from others around his expertise, he expressed concerns about sustainability once he finished residency:

I feel like on the one hand, it’s nice to be there [as the point-person], and to provide other people with the information. On the other hand, it’s shitty because I’m not here forever. […] It almost feels pathetic in the sense that I should be the go-to person as opposed to, a resource being readily available that everybody should be able to access. P11, Psychiatry

This highlights a gap in the current training around caring for trans people in psychiatry, with similar experiences reported from urology residents:

There are some staff that… see the transgender population more frequently but… I’ve been a resident for 3 years. I’ve done a lot of clinic in 3 years and I've only had 3 encounters. I think it's seen more commonly in Montreal. I think they do a lot of their reconstructions in Montreal, I think, but it's not a focus of any one particular staff here. P10, Urology

At the time of conducting the interviews, there were no surgeons in Ontario who did genital reconstruction surgeries such as phalloplasty or vaginoplasty, although there are plans for this to change in the near future.^28^ As such, there are no opportunities in Ontario for residents to learn or observe these surgeries within their own programs. This lack of opportunity perpetuates the problem of a lack of surgical expertise:

No one's comfortable doing a surgery they haven't done hundreds of times, and so until there's an opportunity there for the volume and the training, there's not much taught. P4, Urology

### Navigating experiences with trans patients

A lack of training in clinical skills and medical knowledge were cited as reasons for nervousness and discomfort when providing care to trans patients. This lack of comfort was not due to the patients themselves, but the lack of training in trans care and exposure to trans patients:

For them to ask me to start them on hormone[s], for example, that's something that I may not be prepared to [do] […] I want to, but I may not have the know-how to do it safely. P9, Family Medicine

Simple things you could manage… but when it comes to like reconstructive stuff, I feel like that is not something that… I don't think any residents that graduate from our program would be comfortable [with]. P10, Urology

I’ve done a few surgical follow-ups from top surgery. So I’d be really comfortable doing that. But bottom surgery, I read about but I don’t know how comfortable I would be, to be honest, because I have no experience in […] common post op problems. P6, Family Medicine

While there was some limited exposure to local surgeries on trans patients, these surgeons reportedly had limited training themselves, particularly when handling complications from surgeries performed abroad:

Right now, there's one or two urologists in the city that are sort of helping with complications. But even they don't like doing it, they're not comfortable doing it, and they're not that good at doing it. P4, Urology

Participants also reported feeling nervous in communicating with trans patients as they did not want to contribute to patient distress:

Because there are so many people at different stages in their transition, I do feel that some terms I use could be […] triggering, for these patients. So, I can talk about anatomy, you know, in terms of anatomy “Are you considering surgery?” Could someone say, “Well I don’t need it.” Like would that be offending? And I feel that’s awkward. […] I’m not sure if that’s because maybe transgender patients have had trouble accessing healthcare and I want to be less abrasive or does it speak to my own discomfort? I’m unsure. P5, Family Medicine

It was a bit uncomfortable because I wasn’t as familiar with a lot of the concepts […] like I'm aware that there’s like different terms that people prefer to refer to themselves as and there was one patient … who self-identified as a non-binary, so preferred to be called “it.” But it was really uncomfortable and really unnatural and difficult for me to call this person “it” while I was reviewing with Dr. X. It just seemed very uncomfortable to me. P1, Endocrinology

Inevitably, this lack of experience affects the care that patients receive and their ability to feel safe in the healthcare environment. Participants suggested having trans patients involved in teaching appropriate communication skills for the clinical setting.

Endocrine residents often spoke of benefitting from supportive environments where there were experienced clinicians for them to debrief with following such patient encounters. Reflecting on these experiences and dialogue with experienced mentors helped to build the comfort level and confidence of endocrine residents:

Meaningful exposure is what matters. […] I think the more you see of it, the better you get at it, the more you understand it. Without that experience, I think, … the specifics of transgender care, I would be at the beginning of that learning curve still. P3, Endocrinology

While there was clearly a training gap in which residents did not have enough content teaching about or experience with caring for trans patients, some residents also brought up the idea that having the traits of a “good doctor” enables the ability to care for any patient population, including trans patients:

I haven’t done anything like super remarkable with transgender patients, all I’ve done is show them respect and humility. And it’s just amazing how far that goes with people who have generally not often received that from health care professionals. P7, Psychiatry

### Trans care: Whose job is it?

In addition to a perceived knowledge gap around how to care for trans patients, there was also a lack of clarity around which specialties are responsible for providing care to trans patients. Participants within each specialty held beliefs and assumptions about the training that occurs in other specialties:

I can assume that psychiatry […] may well have something in their curriculum, but I don’t know. Surgery might, but again I don’t know. P2, Endocrinology

I had assumed that they did [mandate that urologists require knowledge on major surgeries], […] I thought only one guy wanted to do it […]. I didn’t realize that no one else was trained. P5, Family Medicine

It was widely acknowledged by the participants that having access to primary care was the gateway to specialist care:

You kind of need a primary care provider to refer you. So if you’re not having really good access to primary care, then you’re probably not getting really good access to special care either. P6, Family Medicine

Family doctors should know who to refer to anytime. P1, Endocrinology

There was a general assumption that endocrinologists are *all* trained in hormone therapy for trans care:

I think in general the people who are focusing on [trans care] are endocrinologists and psychiatrists/psychologists and GPs. P4, Urology

I wonder if every endocrinologist is comfortable with prescribing these medications… But I mean I feel like this is such a common issue that I would hope that most endocrinologists at least have some basic knowledge about this. P7, Psychiatry

However, this was not necessarily the case according to an endocrinology resident:

One of the problems with transgender care is that often the patient will ask the family doctor who doesn't know, or maybe asks another family doctor, or sends to one endocrinologist thinking, you know, 'it's hormones, endocrinologists should probably have this expertise,' and the endocrinologist not only doesn't do it themselves but doesn't know which endocrinologist does do it. P3, Endocrinology

Thus, having access to a primary care physician is a key step in solving the care puzzle. However, there is no guarantee that the recommended specialist will be able to provide adequate and appropriate trans care. These assumptions can contribute to the problem of trans patients facing long wait times to see a specialist who then does not turn out to be skilled in trans care.

## Discussion

Our interviews revealed that family medicine, urology, endocrine, and psychiatry residents at the University of Toronto were not exposed to trans care in a systematic way during their residency. While some endocrine residents had more exposure to trans care than others, like the other specialties, learning often occurred by chance. Interested residents had to seek out elective learning experiences on their own in order to gain adequate experience caring for trans patients.

One main contributing factor to the lack of training opportunities during residency was a lack of faculty with the requisite expertise to teach trans care. The lack of experience with trans patients was problematic in that it contributed to a sense of discomfort for residents, which ultimately affects patient care and experience. Other residents had awkward or challenging experiences when they encountered trans patients, having had little practice communicating with them, lacking knowledge about their care needs and sometimes having been influenced by faculty who also lacked confidence in providing transition-related care (particularly surgical care in urology). Faculty with inadequate training cannot then provide residents with adequate training. This situation contributes to the cycle of trans patients having difficulty accessing healthcare and having uncomfortable encounters when they do. However, our study also showed that residents who had a greater number of clinical experiences and were guided by knowledgeable mentors, were more comfortable in their role caring for trans patients. This is the case in other areas of medicine such as palliative care.^29-31^ This indicates that medical education can play a pivotal role in improving the experiences of trans people seeking healthcare.

Residents in each specialty lacked clarity about the role of the other specialties in providing trans care. Many residents assumed incorrectly that psychiatry residents would have acquired expertise in trans care. This assumption was likely a relic from their prior role as gatekeepers to transition surgeries and hormone therapy.^32^^,^^33^ However, our study revealed that psychiatry residents would only have this capability if they sought elective experiences in this area and this would be challenging given the restraints of intense postgraduate studies. Furthermore, there has been a shift towards primary care physicians playing an increasing role in assessing, initiating, and managing transition-related hormone therapy, and away from psychiatrists having to assess all trans people seeking transition-related care; a movement that has paralleled the de-pathologization of trans people.^1^^,^^33^^,^^34^ However, with the lack of awareness of their potential role in trans care demonstrated by family residents in our study, they would not be able to help further this movement. Another assumption that family and urology residents made in our study was that all endocrinologists would manage hormone therapy, which, unfortunately, is not the case.^13^^,^^35^ Concurrently, there were assumptions that transition-related genital reconstruction surgery was performed at many more sites than were currently available. These false assumptions can unfortunately lead to distressing care delays (due to referrals to the wrong specialists) that amplify the disenfranchisement already felt by many trans people seeking care.

This study elaborates on and provides deeper explanations for the findings in our previous study. ^13^ In that study we found that many family medicine, psychiatry, endocrinology, and urology residents felt unprepared to serve the trans population as they graduate from residency.^13^ The perception that trans care training is structured and effective in other specialties contributes to the idea that “someone else is doing it” which was also a theme our previous study.^13^ Other studies suggest that our health care system is not meeting the increasing demands of the trans population.^2^^,^^4^^,^^6^^,^^7^ Without systemic change to our postgraduate education system, the cycle is likely to continue. Medical societies like the Canadian Psychiatric Association and the Endocrine Society along with prominent medical journals have increasingly recognized the gap in healthcare for the trans population.^1^^,^^36-39^ Our study highlights both the gaps in trans care in the postgraduate education system at one university in Canada’s largest city and that there are residents interested in providing safe and compassionate care for this patient population. Medical education has a significant role to play in addressing the health disparities experienced by this group.

The findings from our study lead us to make the following recommendations:

Systematically embedding trans health education into the curriculum of undergraduate and postgraduate education programs would improve awareness and knowledge around trans care, which in turn would improve the experiences of trans patients. Starting this education in undergraduate programs will hopefully lead to better widespread understanding of gender and trans care issues. Incorporation in postgraduate curriculum will help residents put this into practice. For example, residents should be aware of the concepts of gender identity and gender dysphoria, how the latter is diagnosed and treated, and what other health professionals are involved in trans care. A structural competency approach to this education^40^ would enable residents to appreciate the gaps in care, and the disjointed system of care experienced by trans patients (i.e., being referred, having to wait, lack of knowledge about who is involved in their care, etc.). It would also allow them to understand the inherent biases in our health care system, a system based on the gender binary. Furthermore, residents need to learn how to help trans people navigate the limited resources available for transition-related care. How is transition-related surgery accessed? Who are the health care providers able to provide these procedures? How is the funding accessed? Where are the mental health resources available for counselling? This is an ever-changing but important area which directly impacts on trans patients’ health care, and one in which we found a lot of misunderstanding in our interviews. Utilizing creative solutions, such as virtual groups where clinicians from different disciplines can connect to quickly obtain suggestions on where to refer, or access resources from local experts, could also really improve care and education. Fortunately, some of these networks already exist, e.g. Rainbow Health Ontario’s weekly Trans Mentorship Calls, and British Columbia’s Transgender Care group.

Our results also show that residents interested in trans care often struggle to find meaningful clinical experiences, with knowledgeable mentors to learn from, stemming from the lack of faculty expertise. For example, in Ontario, at the time of our interviews, physicians were not performing transition-related genital reconstruction surgeries. A survey found that endocrinologists rated their competency to care for transgender patients as being low.^35^ In certain programs, there may already be existing faculty with expertise in trans care; however, they have not been given the necessary time in the curriculum. In other programs, there is urgent need to obtain faculty with relevant expertise or otherwise enable current faculty to attain sufficient proficiency. Having more faculty provide trans care in all relevant areas would also mean increased opportunities for the next generation of physicians to learn from experienced teachers.

While we advocate for the inclusion of more specific content around trans care in medical education, there are also aspects of caring for the trans population that require a different approach to training. First, participants in our study provided examples of difficult moments in care that have no straightforward answer but require support. These situations call for carefully constructed, safe opportunities for dialogue, in which contentious and difficult conversations are not avoided during medical education training.^41^ Second, a structural competency framework allows the learner to consider the larger forces contributing to and sustaining disease and health.^40^ Coupled with specific content, these approaches would help learners to better understand the conditions of injustice experienced by trans persons and other marginalized groups, which can ultimately inspire changes in the healthcare system.

By sampling from four different specialties that are important to trans care, our study has shed light on the existing training experiences of residents. Our qualitative approach enabled a rich in-depth exploration of the gaps in knowledge around caring for trans patients. While we utilized a maximum variation strategy, a limitation of the study is that we were not able to sample more within our four specialties; this is a relatively small sample of residents at the University of Toronto. Though the residents we interviewed were near the end of their residency, they still had some time when they may have had further experiences in caring for transgender patients that we did not capture. Further studies could explore specific residency programs in depth or could expand studies to include other specialties likely to encounter trans patients: obstetrics and gynecology, pediatrics, emergency medicine, and plastic surgery.

In summary, our study of the educational experiences and attitudes of family medicine, endocrinology, urology, and psychiatry residents shows a lack of systematic education around trans care. Lack of exposure results in discomfort and misunderstanding around the provision of trans care. We believe medical education can (and should) play a prominent role in addressing the healthcare disparities of this underserved population.
